# The impact of learner background variables on academic burnout in online vs. face-to-face classes among students of Shiraz University of Medical Sciences having English courses

**DOI:** 10.3389/fpsyg.2025.1484760

**Published:** 2025-05-08

**Authors:** Sara Kashefian-Naeeini, Nahid Zarifsanaiey, Manoosh Mehrabi

**Affiliations:** ^1^Department of English Language, School of Paramedical Sciences, Shiraz University of Medical Sciences, Shiraz, Iran; ^2^Department of E-learning, Virtual School, Comprehensive Centre of Excellence for ELearning in Medical Sciences, Shiraz University of Medical Sciences, Shiraz, Iran

**Keywords:** academic burnout, face-to-face learning, learner background variables, online education, medical sciences

## Abstract

**Introduction:**

Academic burnout is a universal phenomenon that has impacted many students in different educational settings. The present study attempted to compare the academic burnout that students of Shiraz University of Medical Sciences (SUMs) faced in online vs. face-to-face environments. It also sought to determine the effects of learner background variables of age, academic achievement, and degree programs on selected students’ academic burnout.

**Methods:**

The power analysis was performed using gpower to determine the required sample size, and 257 participants taking their General English courses were involved in the study. Then, cluster sampling was used to select a random sample from the students taking an English course at SUMS. An online modified version of the Maslach Burnout Inventory (MBI), measuring participants’ academic burnout, was used to represent students’ reported burnout.

**Results:**

A paired samples t-test was performed to compare reported academic burnout in online classes vs. face-to-face environments, indicating significant differences in academic burnout between the two modalities (online vs. face-to-face), with lower levels of burnout reported for online environments. Moreover, a multivariate analysis of variance (MANOVA) was performed to determine the effects of the learner background variables of academic achievement and age on academic burnout, which manifested significant differences in burnout in face-to-face learning environments for students of different age groups. Likewise, it was illuminated that academic achievement influenced students’ burnout in face-to-face environments, as high achievers experienced significantly lower academic burnout than moderate achievers. A robust MANOVA of Pillai’s trace also indicated no significant differences between Bachelor of Science and Professional Doctorate students across the dependent variables of academic burnout in online vs. face-to-face environments.

**Discussion:**

Educators must balance workload, seek regular student feedback about lesson pace and delivery, and avoid overloading students with overdemanding assignments and projects or setting unrealistic goals and expectations. The present study has cast light on the multifaceted nature of burnout and the factors that impact academic burnout in educational settings. It can pave the way for education practitioners to follow appropriate strategies and macro-level policies to mitigate learner burnout.

## 1 Background

Before the outbreak of the COVID-19 pandemic, brick-and-mortar education was prevalent. The pandemic changed the world drastically in different ways. One significant shift occurred in education, where traditional face-to-face instruction was largely supplanted by online classes, prompting a reevaluation of student well-being across learning modalities. For several years, face-to-face education was replaced by online classes in many countries Negahban and Zarifsanaiey (2020). With the comparative control of the pandemic in many parts of the world, universities and other educational settings reopened, and nothing was the same as before since many educators were familiar with online learning and were more eager than before to hold some of their classes online. Therefore, the swing of the pendulum moved toward mixed-mode education. With blended learning, instructors combine in-person instruction with online learning activities. In contrast, with hybrid learning, which offers a flexible learning experience, instructors teach remote and in-person students simultaneously using technological tools such as videoconferencing.

Burnout is a widely known concept in psychological works. According to Maslach and Leiter (2016), it is a three-dimensional structure that incorporates exhaustion, cynicism or depersonalization, and the inefficacy or reduction of personal achievement. Freudenberger (1974) coined the term Burnout to refer to symptoms of fatigue and psychological distress. He examined the deeper causes of burnout and provided effective strategies for relieving its effects. His research emphasized the mental and social elements that contributed to burnout, such as heavy workloads, limited resources, and a lack of support from colleagues and supervisors. Moreover, it set the stage for a more profound understanding of burnout as a multifaceted issue influenced by personal traits and environmental factors. In the past, the definition of burnout was confined to workplace personnel. It was initially held that burnout syndrome influenced only professionals with extensive human interaction (Campos et al., 2012), while it goes well beyond those realms. Over time, the definition above expanded to encompass students. His research emphasized the mental and social elements that contributed to burnout, such as heavy workloads, limited resources, and a lack of support from colleagues and supervisors, setting the stage for a more profound understanding of burnout as a multifaceted issue influenced by personal traits and environmental factors.

A preponderance of studies has been done on teacher or professional burnout. Several studies have, up to now, been conducted on learner burnout in face-to-face learning. With the inception of the COVID-19 pandemic, a few studies started to measure the concept above in online classes. Different studies have been done “on student burnout and the factors affecting it in face-to-face education, and less attention has been paid to the factors affecting burnout in the online environment” (Aria et al., 2024, p. 3989). Moreover, studies comparing the impact of burnout in online vs. face-to-face classes are limited. Rosales-Ricardo et al. (2021) conducted a systematic review examining burnout in university students across 20 studies. The prevalence of each dimension of the burnout syndrome was reported to be 55.4% for emotional exhaustion, 31.6% for cynicism, and 30.9% for academic efficacy. The study also found higher burnout rates in Latin American, Asian, and U.S. students compared to Europeans and identified particularly high burnout among medical, nursing, and engineering students. Additionally, there is a dearth of studies investigating the effect of background variables of age, academic achievement, and degree programs on academic burnout in online vs. face-to-face learning. Thus, the results of the present study may go beyond previous reports, elucidating the influential variables that may impact learner academic burnout. Over and above that, delving into the factors that may affect learner burnout can help instructors and practitioners to get a clearer picture of their students’ abilities and to target their efforts at a more appropriate level. The present study strives to fill the gap and eliminate the preceding sparsity in an EFL context. When educational practitioners are endowed with knowledge of the factors that may influence academic burnout in their learners, they will be disposed to follow more fruitful procedures and include more suitable activities for learners in their face-to-face and online classes.

The detection of burnout and its determining factors at the university level is of paramount importance since, as Toubasi et al. (2023) maintained, the identification of factors that are significantly connected with burnout will pave the way for “both students and educational institutions to implement the strategies needed for the primary and secondary prevention of burnout” (p. 279). Their cross-sectional study at the University of Jordan found high burnout prevalence linked to academic pressure, offering a benchmark for our findings. Some experts scrutinized the impacts of sociodemographic and learner background variables on students’ academic burnout. For example, Lee et al. (2013) investigated age-related differences in academic burnout among Korean students, finding that older students reported higher burnout. Conversely, Mathew (2017) found burnout unrelated to age but tied to stress and lack of support, though we note this source is a thesis and less rigorously peer-reviewed than journal articles.

Similarly, Duru et al. (2014) reported that academic achievement inversely correlated with burnout among Turkish students. In contrast, Yazdi et al. (2018) found no significant link between burnout and demographic variables like age or gender. The level of study is another variable that may influence university students’ burnout. Göldağ (2022) found higher digital burnout among undergraduate students compared to graduates, while Nettam et al. (2018) reported significant differences in stress and burnout between undergraduate and postgraduate students.

Online teaching offers some merits and demerits, and “online platforms have become increasingly important for medical education and training” (Karimian et al., 2024, p. 12). Flexibility, self-paced learning, and networking opportunities are advantages of online modes. However, Hogan and McKnight (2007) reported that online teaching environments cause complexities that could lead to burnout. Likewise, Andrews-Graham (2018) confirmed how shifting to online teaching increased stress levels, leading to emotional exhaustion. Madigan and Curran (2021) who investigated the impact of burnout on academic achievement by conducting a meta-analysis involving over 100,000 students explored whether emotional exhaustion, cynicism, and reduced efficacy—key components of burnout—negatively influenced academic achievement. Their findings emphasized the necessity of addressing student burnout to foster better educational results.

Academic burnout is a negative attitude or behavior toward education that can influence students and teachers at various educational levels and institutions (Navarro-Abal et al., 2018). Hernesniemi et al. (2017) declared that burnout detrimentally affected students’ lives and academic satisfaction, resulting in low academic performance. Mangione et al. (2018) offered solutions, claiming that exposure to literature and arts reduced burnout in medical students. Sunawan et al. (2021) attributed burnout to excessive learning demands, while Naderi et al. (2021) found healthier lifestyles linked to lower burnout, suggesting actionable interventions.

This study attempts to discuss the following research objectives:

- To determine whether or not selected students differ in academic burnout in online vs. face-to-face classes.
- To ascertain whether or not selected participants’ age, academic achievement, and degree programs influence their academic burnout in face-to-face vs. online classes.

Moreover, the following research questions are posed in this study:

- Do selected participants differ in academic burnout in online vs. face-to-face classes?
- Do variables of age, academic achievement, and degree programs influence their academic burnout in face-to-face vs. online classes?

### 1.1 Research variables

In this study, the researchers have investigated the effects of some learner background variables on academic burnout in online vs. face-to-face classes. Thus, academic burnout in online classes, as reported by study participants, is one of the dependent variables, and so is academic burnout in face-to-face classes. One of the learner background variables that may influence academic burnout in different learning environments is the age variable, which is divided into the three categories of below 20, 20 to 22, and 23 and above years old.

Another learner background variable is academic achievement, as revealed by the student’s Cumulative Grade Point Average (CGPA). Iranian versions of the international grading scale were used, with a cumulative grade point average out of 20; students’ CGPAs were classified into three groups: A (17–20), B (14–16.99), and C (13.99 and less). Researchers have claimed that students’ Cumulative Grade Point Average can measure their academic achievement. For instance, researchers like Şimşek et al. (2010) indicated that students’ academic achievement can be operationalized as cumulative grade point average. Likewise, Crede et al. (2015) claimed that students’ GPA can show their academic achievement. Moreover, they used students’ grade point averages to indicate their learning success and academic achievements. Ogundokun et al. (2019) also emphasized that “cumulative grade point average (CGPA) is a system for calculation of GPA scores and is one way to determine a student’s academic performance in a university setting” (p. 154). A third learner background variable is students’ degree programs. We classed the participants into two distinct groups of Professional Doctorate, which included Doctor of Medicine (MD), Doctor of Dental Medicine DMD, and Doctor of Pharmacy (PD), and the Bachelor of Science (BS) group.

**Figure 1:**
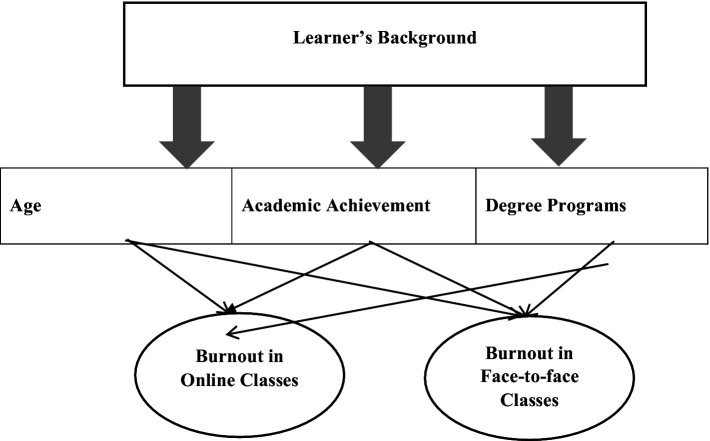
Conceptual model of factors affecting academic burnout.

This study is intended to develop a conceptual model of factors that affect academic burnout in online vs. face-to-face classes, as reported by the study’s participants. Different factors may influence the factors above; this study includes age, academic achievement, and degree programs. These factors are believed to impact learners’ academic burnout in different educational settings. The conceptual framework of the study is shown in Figure 1 below.

## 2 Methodology

### 2.1 Research design

This study employed a cross-sectional design to compare academic burnout among students at Shiraz University of Medical Sciences (SUMS) in online versus face-to-face learning environments and to examine the influence of learner background variables. The present study uses a quantitative approach. Our results will illuminate novel points about academic burnout in online classes compared to face-to-face classes. Moreover, this study sheds light on the impacts of learner background variables on participants’ academic burnout in online and in-person learning environments.

### 2.2 Participants

The study was conducted at SUMS, a public medical university in Shiraz, Iran, from March to June 2024. The study participants were male and female university students taking enrolled in their General English courses. Eligibility criteria included being an active student in a General English course during the study period, with exclusion criteria comprising enrollment in English for Specific Purposes (ESP) courses or failure to complete the study instrument fully. A sample size calculation was performed using GPower software (version 3.1.9.4) with an effect size of 0.2 (small), alpha of 0.05, and power of 0.80, yielding a minimum required sample of 199 participants for a paired samples t-test. The power analysis was performed using GPower to determine the required sample size for the paired samples t-test by comparing the independent groups of students. A desired effect size of 0.2, alpha level of 0.05, and power level of 0.80 were used (Table 1). However, the final sample size was increased to 257 to account for potential attrition (e.g., incomplete responses) and to ensure adequate representation across clusters for multivariate analyses.

**Table 1:** The statistical power analysis of GPower for determining sample size.

MANOVA	Analysis	Input	Output
Means: Difference between two dependent means	*A priori*: Compute the required sample size	Tail(s) = Two Effect size dz. = 0.2 (Small) *α* err prob. = 0.05 Power (1-*β* err prob) = 0.8	Noncentrality parameter *δ* = 2.821347 Critical t = 1.972017 Df = 198 Total sample size = 199 Actual power = 0.801691

GPower version 3.1.9.4, which was used in this study, revealed that a total sample size of at least 199 participants is needed to detect a significant difference between the groups. However, we raised the final count to 257 to address broader analytical and practical issues of availability of participants and the need for greater representativeness. We predicted survey dropout rates as some of participants who are normally involved in a study may choose not to answer questions of the inventory. Inasmuch as classes (clusters) were sampled rather than individuals, a larger sample ensured adequate representation across clusters. Likewise, MANOVA needs larger sample sizes to ensure valid interpretations when assessing interrelated dependent variables.

The cluster sampling method is used. It is a sampling procedure wherein the entire population of interest is divided into groups or clusters (Singh and Masuku, 2014), and a random sample of these clusters is selected. Each cluster must be mutually exclusive, and the clusters must include the entire population. As all the units within a cluster are selected, the sampling procedure in this study is one-stage cluster sampling. Per our sampling procedure, each class is deemed a cluster, and data were collected from every unit in sampled clusters. Thus, all General English course classes offered at SUMs during the data collection period were included in the study. The population of interest comprised 31 intact classes (clusters) which represented students from various academic programs. Using a computer-generated random number list, we selected an appropriate number of these 31 clusters to reach our desired sample size of 257 participants. All general English classes were eligible for selection. However, English for Specific (ESP) classes were excluded from the study. We further ensured data integrity by excluding any cases where participants had not fully completed the Maslach Burnout Inventory (MBI).

### 2.3 Instrument

This study utilized an online version of the Maslach Burnout Inventory-Student Survey (MBI-SS), adapted for this study to measure academic burnout in online and face-to-face contexts. The MBI-SS, validated for Iranian students by Rostami et al. (2014), assesses three dimensions: emotional exhaustion (EE), cynicism (CY), and personal accomplishment (PA). The questionnaire included 44 items, 22 of which were about the burnout students faced in online classes, and the rest were about the burnout students encountered in face-to-face classes. Each dimension was measured using a 7-point Likert scale (0 = never, 6 = every day), with higher EE and CY scores and lower PA scores indicating greater burnout.

We used a modified version of the original MBI which was especially adapted for academic contexts to determine academic burnout rather than occupational burnout. This version allowed greater flexibility in creating equivalent versions for online and face-to-face learning environments. Contextual wording adjustments were made and the original MBI items were translated into Persian and then carefully reviewed for relevance to both online and face-to-face academic settings. To enable direct comparison between online and face-to-face burnout, the two sets of items (online vs. face-to-face) were kept parallel in content except for the explicit mention of the learning environment. This ensured measurement consistency across modes while considering contextual differences (e.g., digital fatigue in online settings vs. physical exhaustion in face-to-face).

The present study collected age (categorized into below 20, between 20 and 22, 23 and over), academic achievement (operationalized as Cumulative Grade Point Average, CGPA), and degree programs (classified as Bachelor of Science vs. Professional Doctorate) as part of the sociodemographic data within our survey instrument. These variables were essential to our analysis of academic burnout across learning modalities.

#### 2.3.1 Reliability and validity procedures

Cronbach’s alpha was calculated to assess the internal consistency of the adapted MBI-SS. According to Trizano-Hermosilla and Alvarado (2016), “the Cronbach’s alpha is the most widely used method for estimating internal consistency reliability. This procedure has proved very resistant to the passage of time, even if its limitations are well documented and although there are better options as omega coefficient or the different versions of glb, with obvious advantages especially for applied research in which the items differ in quality or have skewed distributions” (p. 1).

The alpha index for the whole questionnaire was 0.94, indicating high internal consistency. Thus, the high reliability index of the adapted inventory made it particularly suitable for our study as it allowed us to maintain measurement consistency across both learning environments. Even though Maslach’s burnout inventory has previously been checked for validity and validated in multiple ways, it was validated again in this study by several professors and experts in the field. Two SUMS professors with expertise in psychology and education, translated the English version of the inventory, followed by back-translation to ensure equivalence. Then, the items were reviewed by a number of professors at SUMS to check the content of the inventory and mark appropriate and inappropriate statements for cultural appropriateness and academic relevance, which led to minor modifications that maintained the original constructs of emotional exhaustion, depersonalization, and reduced personal accomplishment.

### 2.4 Procedure

The participants were selected from two degree programs: Professional Doctorate (e.g., Medicine, Dentistry, Pharmacy) and Bachelor of Science. The study followed strict ethical standards to guarantee the rights and confidentiality of participants. Ethical approval was obtained from the Ethics Committee of Shiraz University of Medical Sciences (SUMS) under code 29471. Participants were thoroughly informed about procedures, and their right to withdraw without facing any consequences. No personal identifying details (such as names or email addresses) were collected, reducing the likelihood of tracking individual participants. Moreover, access to stored data remained strictly limited to authorized research team members on secure server. The purposes of the study were explicated to the participants, via an email invitation that included an informed consent form outlining voluntary participation, study aims, and confidentiality measures. Participants provided consent electronically by clicking an ‘agree’ button before accessing the survey, with data stored on a password-protected server accessible only to the research team. Moreover, as the data collected were used solely for this study, they were invited to email the researchers later if they were interested in the study results. An online modified version of the MBI-SS, which measures participants’ views about their academic burnout, was administered to students via a secure online platform (Google Forms) with instructions to respond based on their experiences in General English courses. The Participants were instructed to answer the questions as carefully as possible and not to leave any items unanswered. A brief sociodemographic questionnaire collected data on age, gender, degree program, and Cumulative Grade Point Average (CGPA) immediately before the MBI-SS.

In the context of our study, it is important to differentiate between ‘dropouts’ and ‘missing data’. Throughout the survey process, we experienced a number of dropouts, as some participants decided not to complete the questionnaire. However, it is essential to note that the design of the questionnaire ensured that there was no missing data among the responses we collected. The questionnaire was structured in such a way that participants could not finalize their submissions unless all items were answered. This design feature effectively eliminated the possibility of missing data, as any participant who attempted to submit an incomplete questionnaire was prompted to return to the unanswered items. As a result, while some participants did not finish the questionnaire, every participant who completed the questionnaire provided responses to all items.

### 2.5 Data analysis

Descriptive statistics such as frequencies, means, and standard deviations were used in the present study. A paired-sample t-test was used as a parametric test to compare the means of two measurements (burnout in online vs. face-to-face classes) taken from the same participants. Since burnout as a dependent variable has three dimensions, our data were also analyzed through multivariate analysis of variance (MANOVA). This statistical procedure allows the comparison of different groups of participants on several different but related dependent variables. The major difference between MANOVA and analysis of variance (ANOVA) is that MANOVA can examine the differences among mean values of many variables and reduce the probability of Type 1 error. Therefore, due to its numerous advantages over ANOVA and many other statistical techniques, the statistical technique of MANOVA helps to measure the effects of age, academic achievement (as shown by Cumulative Grade Point Average or CGPA), and degree programs as independent variables on selected participants’ academic burnout in online vs. face-to-face classes (dependent variables). To address potential bias, incomplete responses (<2% of cases) were excluded via listwise deletion, and sensitivity analyses confirmed results were robust to this approach. The Statistical Package for Social Sciences (SPSS) version 26 was used for all measurements.

## 3 Results

Our study comprised 257 participants from Shiraz University of Medical Sciences, all of whom were enrolled in General English courses. Participants were categorized by age, with 44% under 20 years, 45.5% between 20 to 22 years, and 10.5% aged 23 and above. In terms of academic performance, 32% of the students achieved a high cumulative grade point average (CGPA) ranging from 17 to 20, 45% were identified as moderate achievers with CGPAs between 14 and 16.99, and 23% were classified as low achievers with CGPAs of 13.99 or below. Moreover, participants were divided by their degree programs, with 34% following Bachelor of Science degrees and 66% enrolled in Professional Doctorate programs, including Medicine, Dentistry, and Pharmacy.

The following first section is dedicated to the results of data analysis through paired-sample t-test and Wilcoxon Signed Ranks Tests, and the second section deals with the results related to multivariate analysis of variance. To address the research question under the categories of the dependent variables and independent variables (age, academic achievement, and degree programs), we analyzed the data using Multiple Analysis of Variance (MANOVA).

### 3.1 Research question 1. Do selected participants differ in their academic burnout in online vs. face-to-face classes?

A paired samples t-test compared students’ reported academic burnout in online and face-to-face classes. Table 2 illustrates the descriptive statistics and compares academic burnout levels in online vs. face-to-face classes.

**Table 2:** Paired sample statistics for the comparison of students’ burnout in online and face-to-face classes.

Burnout	Mean	N	Std. deviation	t(df)	*p*-value
online	1.6332	257	1.24678	−2.861(256)	0.005
face-to-face	1.9763	257	1.43372		

The results indicated that there was a significant difference in academic burnout between the two modalities, with lower levels of burnout reported in online classes in comparison with face-to-face classes (*p* = 0.005). This finding suggested that students experience greater academic burnout in face-to-face learning environments compared to online settings. A series of Wilcoxon Signed Ranks Tests were carried out to evaluate the differences in academic burnout in online vs. face-to-face classes across the three dimensions of academic burnout—Emotional Burnout (EB; Online EB vs. face-to-face EB), Depersonalization (Online depersonalization vs. face-to-face depersonalization), and Personal Accomplishment (PA; Online PA vs. face-to-face PA). The results are given in Table 3. Given that three comparisons were made, a Bonferroni correction was applied, setting the significance level at *α* = 0.05/3 = 0.0167.

**Table 3:** Wilcoxon signed ranks test results comparing online and face-to-face academic burnout dimensions.

Dimension	Median (Online)	Median (Face-to-Face)	Z	Sig
Emotional Burnout	1.56	2.56	−4.589	0.001
Depersonalization	1.20	1.00	−0.377	0.707
Personal Accomplishment	1.38	1.25	−1.675	0.094

A significant difference in Emotional Burnout scores was revealed by the Wilcoxon Signed Ranks Test, illuminating that the participants had higher burnout levels in face-to-face classes as opposed to online classes (*p* < 0.001). This finding meets the Bonferroni-adjusted significance level. The results of the two other burnout dimensions were not significant for depersonalization and for personal accomplishment.

### 3.2 Research question 2. How do learners’ background factors influence academic burnout in online vs. face-to-face classes?

#### 3.2.1 Age and academic burnout

Levene’s and Box’s M tests were insignificant for the dependent variables of academic burnout in online vs. face-to-face classes. Appendix Table A.2 shows the findings of Box’s M test. These results allowed the multivariate analysis of variance to be used to analyze the level of differences between students of different age groups in relation to the dependent variables.

Data obtained from students of different age groups revealed that the error variances of the dependent variables were equal across groups, and the assumptions of homogeneity of error variances were not violated among the groups. Therefore, the assumption of equal variances was met for the following analyses, and MANOVA could be conducted. Appendix Table A.2 displays the results of Levene’s test of equality of variance.

A MANOVA mixed-group design (group × measures) was performed to determine the effect of age on academic burnout in online vs. face-to-face classes. The multivariate test was performed on the data at the 0.05 significance level. Findings from the multivariate test of Wilks’s Lambda showed a highly significant age main effect [Wilks’ *Λ* = 0.942, *F* (4, 506) = 3.869, *p* = 0.004] on students’ academic burnout. The detailed results of academic achievement by age are shown in Table 4 in section 3.2.3, together with the MANOVA results of other independent variables in the study.

**Table 4:** MANOVA for academic burnout by age, academic achievement, and degree programs.

Effect	Value	F	Hypo df	Err df	Sig	Par Eta Sq	Power
Age Wilk’s Lambda	0.942	3.869	4	506	0.004	0.03	0.898
Academic Achievement Wilk’s Lambda	0.948	3.450	4	506	0.009	0.027	0.857
Degree Programs Pillai’s Trace	0.997	0.424	2	245	0.655	0.003	0.848

The univariate analysis provides the proportion of variance in the dependent variables that can be attributed to different levels of an independent variable (i.e., the different levels of the independent variable of age: below 20, between 20 and 22, 23 and over). Results of the univariate analysis of variance are shown in Appendix Table A.3. A significant main effect of age was found for academic burnout in both online classes and face-to-face classes.

Since the univariate test results were statistically significant, statistical significance was put at an alpha level of *p* < 0.05. Scheffe *post-hoc* multiple comparison was used to determine where the differences between means existed. It was found that there were significant differences in academic burnout in face-to-face classes among students who were below 20 years of age and those who were between 20 and 22 (*p = 0.012* < 0.05). The results are displayed in Table 5.

**Table 5:** The Results of the Scheffe *post-hoc* test for students of different age.

Dependent variable	(I) Age	(J) Age	Mean difference (I-J)	Std. error	Sig.
Burnout in online classes	Below 20	Between 20 to 22	0.1542	0.16315	0.640
		23 and over	0.6524	0.26496	0.050
	Between 20 to 22	Below 20	−0.1542	0.16315	0.640
		23 and over	0.4982	0.26409	0.171
	23 and over	Below 20	−0.6524	0.26496	0.050
		Between 20 to 22	−0.4982	0.26409	0.171
Burnout in face-to-face classes	Below 20	Between 20 to 22	−0.5580^*^	0.18636	0.012
		23 and over	−0.5116	0.30266	0.242
	Between 20 to 22	Below 20	0.5580^*^	0.18636	0.012
		23 and over	0.0464	0.30166	0.988
	23 and over	Below 20	0.5116	0.30266	0.242
		Between 20 to 22	−0.0464	0.30166	0.988

*The mean difference is significant at the 0.05 level.

Descriptive statistics on academic burnout in online vs. face-to-face classes indicated that for burnout in online classes, the highest academic burnout means were obtained by the youngest students (those below 20 years of age), and the lowest burnout means were gained by the oldest students (those who were 23 and above). However, for burnout in face-to-face classes, the lowest vector of means was obtained by students of the youngest group. The means and standard deviations for academic burnout of the different age groups are shown in Table 6.

**Table 6:** Descriptive statistics for academic burnout among students of different age groups.

Mode	Age	Mean	Std. deviation	N
Online	Below 20	1.7719	1.25586	113
	Between 20 to 22	1.6177	1.27815	117
	23 and over	1.1195	0.92959	27
	Total	1.6332	1.24678	257
Face-to-face	Below 20	1.6685	1.35471	113
	Between 20 to 22	2.2265	1.40067	117
	23 and over	2.1801	1.68642	27
	Total	1.9763	1.43372	257

#### 3.2.2 Academic achievement and academic burnout

The next background variable was academic achievement. Box’s M test was conducted to check the homogeneity-of-co-variance-matrices of the dependent variables of academic burnout in online and face-to-face classes, which yielded a nonsignificant result (*F* = 1.086, *p* = 0.368 > 0.05). This suggested that there were equal-error variance and co-variance matrices. Levene’s test of homogeneity of variance was not statistically significant for the dependent variables, either. These two results allowed the MANOVA to be used to analyze the differences among university students of different academic achievement groups concerning academic burnout in online vs. face-to-face settings. The results of Box’s and Levene’s tests are shown in Appendix Tables A.4, Appendix Table A.5, respectively.

Their reported burnout means were considered for calculating the differences among students with different academic achievements (high academic achievement, moderate academic achievement, and low academic achievement). Students who had the highest academic achievement, as shown by their CGPAs, reported experiencing the highest academic burnout in online classes. In contrast, the students with the lowest CGPAs had the lowest burnout means both in online classes (M = 1.14, SD = 1.16) and face-to-face classes (M = 1.65, SD = 1.27). Findings from the multivariate test of Wilk’s Lambda yielded a significant Wilks’ *Λ* = 0.948, *F* (4, 506) = 3.45, and *p* = 0.009 for academic burnout in online and face-to-face classes among students of different academic achievement which are shown in Table 4 in the next section.

Following the initial MANOVA, a univariate analysis of variance was run. For academic burnout in online classes, a nonsignificant main effect of academic achievement was revealed; however, for academic burnout in face-to-face classes, the results indicated a significant academic achievement main effect (*F* = 5, *p* = 0.007, η^2^ = 0.038). Scheffe *post-hoc* test was used, and statistical significance was put at an alpha level of *p* < 0.05. It was illuminated that there were significant differences in academic burnout in face-to-face classes among students with high academic achievement and those with moderate academic achievement; students with high academic achievement experienced significantly lower academic burnout than those with moderate academic achievement (*p* = 0.038 < 0.05). The results of the univariate analysis are displayed in Appendix Table A.6, and the *post-hoc* test results are presented Appendix Table A.7.

#### Degree programs and academic burnout

The next variable that was considered in this study was the variable of degree programs. All bachelor’s degree students were categorized as Bachelor of Science, while the students of Medicine, Dentistry, and Pharmacy were considered Professional Doctorate students. Significantly, Levene’s test of homogeneity of variances for burnout in online classes prompted using Pillai’s trace for a robust MANOVA, which enhances the credibility of the results and methodological rigor. Pillai’s trace was nonsignificant, which indicated that there were no significant differences between Bachelor of Science and Professional Doctorate students across the dependent variables of burnout in online and face-to-face classes, Pillai’s Trace = 0.003, *F*(2, 254) = 0.424, *p* = 0.655. The results of univariate analysis also revealed nonsignificant main effects of degree programs on academic burnout in both online classes (*F* = 0.838, *p* = 0.361, η^2^ = 0.003) and face-to-face classes (*F* = 0.018, *p* = 0.893, η^2^ = 0.000). Table 4 outlines the results of Pillai’s Trace as a robust MANOVA for academic burnout by degree programs (the last row of results). Moreover, this table clarifies that the multivariate test of Wilk’s Lambda yielded a significant Wilks’ *Λ* for academic burnout in online and face-to-face classes among students of different age and academic achievement (as shown in the first two rows of the table).

## 4 Discussion

This study intended to determine whether or not selected students differ in academic burnout in online vs. face-to-face classes. It also sought to specify whether or not the participants’ age, academic achievement, and degree programs influenced their reported academic burnout in online vs. face-to-face classes. It was revealed that students experienced greater academic burnout in face-to-face learning environments compared to online settings, with highest burnout level for the dimension emotional burnout. A plausible explanation is that face-to-face classes encompass direct interaction with lecturers and classmates, which can result in social pressure, emotional fatigue and burnout. Attending in-person classes may cause more fatigue and a greater workload for students. Moreover, online learning provides more flexibility and offers better chances for self-paced learning, not to mention greater independence in how students manage their time, all of which can lead to decreased burnout.

Comparatively, Andrews-Graham (2018) found that the shift from online format to in-person classes caused participants to experience lingering effects of burnout. Moreover, Navarro and Shoemaker (2000) reported some of the advantages of using online education by stating that in an online setting, shy and easily intimidated students are provided a sense of anonymity in which they can take part more freely with fewer challenges in communication which can lead to greater confidence and decreased burnout. Our results are inconsistent with those of Mosleh et al. (2022), who discouraged the use of online teaching by reporting that a sudden shift to online classes can lead to burnout, exhaustion, and frustration, and Hunt et al. (2023), which confirmed that the transition to exclusive online learning brought about higher levels of burnout in comparison to in-person learning. Hogan and Mcknight (2007), who investigated the prevalence of burnout symptoms, also affirmed that a stigma is attached to online instruction, which makes it a main stressor that can lead to burnout. Thus, as an important component of online courses, instructors should model effective participation and collaboration, resolve technical problems, and create a safe learning environment for learners. Tagoe (2012) found a solution to this problem by advocating blended learning in which university students receive a combination of online and face-to-face teaching. Rosales-Ricardo et al. (2021) reported that out of the dimensions of burnout, emotional burnout had the highest prevalence, which supports our findings.

The youngest students (those below 20) had significantly lower burnout in face-to-face classes than older students. However, they reported the highest burnout in online classes. These youngest students spent most of their high school years during the coronavirus pandemic years, with the bitter experiences of the prevalent disease and the obligatory online classes. Participants of other age groups were more mature then and perhaps had greater tolerance (Zarifsanaiey et al., 2022). Thus, the fact that the youngest students had the highest academic burnout in online classes and the lowest in face-to-face classes may be attributed to their previous experiences with online classes at the time of the pandemic. Likewise, Aria et al. (2024), by referring to the closure of educational centers and the shift to online education, reported that a rapid and unplanned transition from face-to-face education to online education combined with concerns about coronavirus infection led to stressful learning environments and the risk of increasing burnout among students. The COVID-19 pandemic, which harmfully influenced the transfer of instruction, necessitated some changes and adjustments to the modes of teaching, which, as Coman et al. (2020) emphasized, was challenging as the adaptations to the new setup were difficult since improved preparation together with suitable internet access was essential for the online experience. Moreover, students and teachers were not ready for an entirely online experience. Therefore, the main challenges that students encountered were, according to Coman, problems of accessibility, connectivity, lack of appropriate devices, and social issues shown by the lack of communication and interaction with instructors and peers, all of which may lead to academic burnout.

Adopting the online learning technique has exposed students to a large scope of difficulties (Quilon and Kurniawan, 2023), which should be alleviated to hold online classes successfully. Conducting effective online classes necessitates several infrastructures as essential elements to facilitate a productive educational experience for students and instructors. One of them is the technology infrastructure, which includes a learning management system (LMS) used in medical sciences universities in Iran to organize courses and present materials. A stable internet connection is also a necessity to hold online classes. In addition, there are some software and hardware requirements together with content infrastructures such as comprehensive course materials and organized resources for students, not to mention the support systems to remove technical problems. Likewise, Cable and Cheung (2017), by adding an eighth principle to what is needed to hold online teaching, reported that encouraging student-faculty contact, enhancing collaborative learning, improving active learning, providing quick feedback, emphasizing time on task, setting and communicating high expectations, respecting different talents and ways of learning and applying technology are necessary in an online environment. Lack of these principles may aggravate students’ peace of mind and cause academic burnout.

Our results are consistent with those of Lee et al. (2013) who indicated that academic burnout increased gradually as age increased since younger participants exhibited lower levels of burnout compared to older ones. Thus, they suggested a correlation between age and burnout levels, claiming that as students progress through their academic journey, they may encounter challenges that contribute to heightened feelings of burnout. Our findings also align with Muzafar et al. (2015) results which demonstrated that age was significantly associated with burnout in that older medical students were more burnt out than younger ones. However, what we found stands in contrast to those of some experts in the field which emphasized that age was not associated with burnout (for example, Yazdi et al., 2018; Santen et al., 2010; Prins et al., 2007).

In our study, academic achievement, as represented by students’ CGPA, had impacts on students’ academic burnout and students with high academic achievement experienced significantly lower academic burnout than those with moderate academic achievement in face-to-face environments. This may stem from the fact that high-achievers are usually goal-oriented and set their academic goals in a way to meet their objectives. They also employ suitable coping strategies, have great engagement, use enriched supportive environments, available resources and effective time-management techniques in managing their tasks and assignments all of which can prevent academic burnout. Moreover, high-achieving students usually have greater self-efficacy in their mettles to excel in academic pursuits which results in reduced stress and lower burnout.

Our finding is in contrast to that of Daud et al. (2020) which reinforced that CGPA was not associated with burnout and that of Duru et al. (2014) which demonstrated that academic achievement was negatively associated with most of the dimensions of burnout. Our outcomes also differ from that of Lyndon et al. (2017) which highlighted that burnout was connected with lower academic achievement; however, it is in line with Toubasi et al. (2023) findings which reported that GPA was significantly associated with burnout and its constituents and with Madigan and Curran (2021) research which linked higher burnout levels to lower academic performance.

Though all the variables of our study yielded statistically significant results, there was one factor which showed non-significant results and that was the variable of degree programs. No significant differences were reported across the dependent variables of burnout between Bachelor of Science and Professional Doctorate students in online vs. face-to-face environments. This could be explained by the similarity of academic experience between PD and BS students as they may have comparable educational challenges, unparalleled stressors, identical support systems and academic services. Moreover, corresponding external factors may influence their learning experiences in online and in-person environments. Students in both groups may employ similar coping strategies to handle stress and burnout which may reduce the difference leading to the observed results. Thus, academic burnout might originate from different stressors that are common to all our participants regardless of their degree programs. The findings of our research are at odds with that of Nettam et al. (2018) which demonstrated significant differences in stress and burnout levels between the postgraduate and undergraduate since postgraduate students had more intense academic demands than undergraduate students, leading to higher levels of stress and burnout. Moreover, our results stand in contrast to that of Göldağ (2022) which confirmed that burnout levels are higher among students studying at the undergraduate level than those studying at the graduate level.

## 5 Implications

By illustrating the fact that academic burnout was significantly higher in face-to-face classes than online classes, this study provides a practical roadmap for educators striving to refine classroom dynamics and student outcomes (Agustina et al., 2019). University instructors should follow a multifaceted approach in their face-to-face classes which diminishes different stressors and augments a healthier learning environment. To reduce burnout in the aforementioned learning environment, instructors are required to balance workload, seek regular student feedback about the pace and delivery of lessons and avoid overloading university students with over demanding assignments and projects or setting unrealistic goals and expectations. Educators can set more flexible deadlines for the completion of tasks by their students and use blended learning models in which online and traditional in-person learning are integrated (Krejcie and Morgan, 1970; Maslach et al., 1997). As students reported greater degrees of burnout in traditional face-to-face classes than online classes, lecturers should hold some of their classes online in addition to their in-person classes so that the blended learning approach can help them use different multimedia and interactive tools to boost student engagement and provide more dynamic learning environments which uses the advantages of both learning modes and ends in a more flexible personalized learning experience. That our participants reported significantly higher emotional burnout scores in face-to-face classes in comparison with online environments underlines the necessity of addressing emotional burnout through interventions aimed at providing better emotional support and stress management resources for students in a face-to-face setting.

An interpretation of findings revealed that age significantly impacted academic burnout and youngest students had the highest burnout in face-to-face classes and the lowest burnout in online environments (Maslach et al., 2009; Salmela-Aro et al., 2009). Therefore, it is necessary that educators consider age differences while planning their curricula for face-to-face classes. Some supportive services, stress management workshops and mentorship programs can be held for the aforementioned age group. Moreover, since students with high academic achievement had significantly lowest burnout in comparison to moderate achievers in face-to-face settings, there is a need for tailored approaches in which different academic achievement levels are regarded while implementing programs that mitigate academic burnout, especially in face-to-face classes. Educational policy makers had better run some counseling services and peer support programs for moderate and low achievers.

The insignificant effects of degree programs on academic burnout among PD and BS students imply that challenges connected with academic burnout are pervasive in different fields of study and that both groups experienced similar levels of burnout, irrespective of their program (Pamungkas and Nurlaili, 2021; Oloidi et al., 2022). Thus, measures to address burnout may focus on all student population similarly and mental help services and stress management workshops can assist both groups in moderating their burnout. Professional development sessions can also be arranged for lecturers to learn the most effective ways to help students of different degree programs diminish burnout both in online and in-person environments.

Our research was limited to students of different fields of study from one of the universities of medical sciences. This research can be expanded to include students from diverse populations, including students from different universities, regions, races and countries. Cross-cultural studies can also provide deeper insights into learner burnout issues in different cultural and educational settings and make cross-cultural comparisons feasible. Moreover, our study exclusively included the burnout that was experienced by university learners in online and in-person settings. Some studies could be conducted to assess the burnout of university instructors, chairpersons, deans and chancellors of universities and to examine the interrelationships of those measures with learner burnout.

This study encompassed the variables of age, academic achievement and degree programs. Studies in which the effects of the different sociodemographic factors of gender, cultural background, profession, marital and socioeconomic status and parents’ education can also lead to informed policy making. Moreover, some future research works which seek the impacts of learner background variables such as study habits, coping strategies, career aspirations, learning styles and strategies and personality traits such perfectionism on academic burnout can lead to tailored interventions and improved academic outcome in online and in-person settings. Burnout does not occur straight away. On the contrary it is a condition which happens due to the cumulative interaction of different factors in the developmental process (Özhan and Yüksel, 2021) in different educational settings. Therefore, the correct recognition and timely acknowledgement of academic burnout together with its’ intervening factors will help educational practitioners follow serious commitment to amend university students’ condition and to resort to the best educational mode that may diminish academic burnout in students.

## 6 Conclusion

Academic burnout is a state which is characterized by the exhaustion that university students encounter because of the stress which is induced by their academic responsibilities. It can be experienced not only in in-person settings but also in online environments. Thus, using a standard research inventory, university students’ reported academic burnout was compared in online classes vs. face-to-face classes. Moreover, the effects of the learner background variables of age, academic program, and degree programs were explored on academic burnout in the two learning environments. With higher levels of burnout experienced in face-to-face classes, it is recommended that educators use blended learning modes in which both online and in-person classes are integrated. Sticking to the traditional face-to-face classes exclusively and keeping online classes on the periphery while disregarding the numerous benefits that online learning may offer leads students to ever-increasing levels of burnout. While degree programs did not impact learner burnout, age and academic achievement exerted significant effects on students’ academic burnout in face-to-face environments, with youngest and highest academic achievers showing lowest levels of academic burnout.

Though much research has focused on burnout in face-to-face classroom settings, there remains a notable gap in studies comparing it to online environments. This study fills this critical gap in burnout literature by providing direct comparison between online and face-to-face learning environments among medical students, an area that has received limited scholarly attention. Contrary to expectations, results indicate substantially reduced burnout in online classes, especially regarding emotional burnout. Fresh discoveries emerge regarding the variable impacts of ‘age’ and ‘academic achievement’ on burnout manifestations, revealing that younger students experience greater online burnout, while high achievers cope better in traditional face-to-face classrooms. Interestingly, burnout trends proved consistent across all disciplines, challenging existing assumptions that stress levels differ by academic program.

This study positions burnout research within contemporary digital education frameworks, particularly in medical training where stress levels are historically high. By identifying consistent burnout patterns across disciplines and the beneficial impacts of online learning for particular subgroups, it encourages reevaluation of long-held views about educational approaches in demanding academic settings. Likewise, this investigation underscores the need for policymakers to note the multifaceted nature of burnout and to reconsider the effects of learner background variables on academic burnout. Accordingly, the implications of this study highlight the necessity of enhancing support systems and curricula, lowering workloads especially in face-to-face classes and developing coping strategies. Likewise, professional development programs and adequate training sessions should be organized for instructors on the reevaluation of previous procedures, identification of burnout signs in students, and the use of effective strategies to help students moderate their academic burnout on their way to self-reflection and personal growth. Our results can also pave the way for determining the effects of interventions such as holding counseling sessions for students, running workshops and providing peer support which can alleviate academic burnout among university students in different learning environments.

## Data Availability

The raw data supporting the conclusions of this article will be made available by the authors, without undue reservation.

## Supplementary material

The Supplementary material for this article can befound online at: https://www.frontiersin.org/articles/10.3389/fpsyg.2025.1484760/full#supplementary-material
