# Supplementary material for: The impact of learner background variables on academic burnout in online vs. face-to-face classes among students of Shiraz University of Medical Sciences having English courses
Source: Front Psychol. 2025 May 8;16:1484760. doi: 10.3389/fpsyg.2025.1484760 (PMC12095369; doi:10.3389/fpsyg.2025.1484760)
Supplement: Supplementary file 1 [file Table_1.docx]

**Appendix**

Table A.1 shows the findings of Box's M test, which was used to determine the homogeneity of covariance matrices of the dependent variables. These results allowed the MANOVA to be used to analyze the level of differences between students of different age groups in relation to the dependent variables: Box's M = 11.609, F(6, 43087) = 1.89, p=0.077>.05.

**TABLE A.1 Box’s test of equality of covariance matrices for the age variable**

| **Box’s M** | **F** | **df1** | **df2** | **Sig.** |
| --- | --- | --- | --- | --- |
| 11.609 | 1.898 | 6 | 43087.475 | .077 |

**TABLE A.2 Levene's test of equality of error variances for the age variable**

|  | **Leven Statistic** | **df1** | **df2** | **Sig.** |
| --- | --- | --- | --- | --- |
| Burnout in Online Classes | 2.093 | 2 | 254 | .125 |
| Burnout in face-to-face Classes | 1.309 | 2 | 254 | .272 |

Table A.3 presents the results of the univariate analysis for the age variable. The alpha was set at the .05 level, and the analysis revealed a significant main effect of age on academic burnout in both online classes (F = 3.048, p = .049, η^2^ = .023) and face-to-face classes (F = 4.796, p = .009, η^2^ = .036).

**TABLE A.3 Univariate analysis (Tests of between-subjects effects) for the age variable**

| **Source** | **Sum of Square** | **df** | **Mean Square** | **F** | **Sig** | **Par Eta**  **Sq** |
| --- | --- | --- | --- | --- | --- | --- |
| Burnout in Online  Classes | 9.327 | 2 | 4.663 | 3.048 | .049 | .023 |
| Burnout in Face-to-face  Classes | 19.148 | 2 | 9.574 | 4.796 | .009 | .036 |

**TABLE A.4 Box’s test of equality of covariance matrices for the variable of academic achievement**

| **Box’s M** | **F** | **df1** | **df2** | **Sig.** |
| --- | --- | --- | --- | --- |
| 6.667 | 1.086 | 6 | 25467.571 | .368 |

**TABLE A.5 Levene's test of equality of error variances for the variable of academic achievement**

|  | **Leven Statistic** | **df1** | **df2** | **Sig.** |
| --- | --- | --- | --- | --- |
| Burnout in Online Classes | .501 | 2 | 254 | .606 |
| Burnout in face-to-face Classes | .361 | 2 | 254 | .698 |

**TABLE A.6 Univariate analysis (Tests of between-subjects effects) for the variable of academic achievement**

| **Source** | **Sum of Square** | **df** | **Mean Square** | **F** | **Sig** | **Par Eta**  **Sq** |
| --- | --- | --- | --- | --- | --- | --- |
| Burnout in Online  Classes | 5.858 | 2 | 2.929 | 1.897 | .152 | .015 |
| Burnout in Face-to-face  Classes | 19.934 | 2 | 9.967 | 5 | .007 | .038 |

**TABLE A.7 The results of Scheffe post-hoc test for students of different academic achievement**

| **Dependent Variable** | **(I) Academic Achievement (CGPA)** | **(J) Academic Achievement (CGPA)** | **Mean Difference**  **(I-J)** | **Std. Error** | **Sig.** |
| --- | --- | --- | --- | --- | --- |
|  |  |  |  |  |  |
| Burnout in  Online Classes | 17 to 20 | 14 to 16.99 | .0341 | .16392 | .979 |
|  |  | Under 14 | .5510 | .28566 | .158 |
|  | 14 to 16.99 | 17 to 20 | -.0341 | .16392 | .979 |
|  |  | Under 14 | .5170 | .29258 | .212 |
|  | Under 14 | 17 to 20 | -.5510 | .28566 | .158 |
|  |  | 14 to 16.99 | -.5170 | .29258 | .212 |
| Burnout in  face-to-face  Classes | 17 to 20 | 14 to 16.99 | -.5521^*^ | .18627 | .013 |
|  |  | Under 14 | .1120 | .32460 | .942 |
|  | 14 to 16.99 | 17 to 20 | .5521^*^ | .18627 | .013 |
|  |  | Under 14 | .6641 | .33247 | .138 |
|  | Under 14 | 17 to 20 | -.1120 | .32460 | .942 |
|  |  | 14 to 16.99 | -.6641 | .33247 | .138 |

* The mean difference is significant at the .05 level
